# Macrofilaricidal Efficacy of Repeated Doses of Ivermectin for the Treatment of River Blindness

**DOI:** 10.1093/cid/cix616

**Published:** 2017-07-19

**Authors:** Martin Walker, Sébastien D S Pion, Hanwei Fang, Jacques Gardon, Joseph Kamgno, Maria-Gloria Basáñez, Michel Boussinesq

**Affiliations:** 1 Department of Pathobiology and Population Sciences and London Centre for Neglected Tropical Disease Research, Royal Veterinary College, Hatfield; 2 Department of Infectious Disease Epidemiology and London Centre for Neglected Tropical Disease Research, Imperial College London, United Kingdom; 3 UMI233-TransVIHMI, Institut de Recherche pour le Développement, INSERM U1175, University of Montpellier, France; 4 Department of Microbiology and Molecular Medicine, University of Geneva Medical School, Switzerland; 5 Hydrosciences Montpellier, Institut de Recherche pour le Développement, France; 6 Centre for Research on Filariasis and Other Tropical Diseases; 7 Faculty of Medicine and Biomedical Sciences University of Yaoundé I, Yaoundé, Cameroon.

**Keywords:** onchocerciasis, river blindness, ivermectin, macrofilaricide, multiple dose

## Abstract

**Background:**

Mass drug administration (MDA) with ivermectin is the cornerstone of efforts to eliminate human onchocerciasis by 2020 or 2025. The feasibility of elimination crucially depends on the effects of multiple ivermectin doses on *Onchocerca volvulus*. A single ivermectin (standard) dose clears the skin-dwelling microfilarial progeny of adult worms (macrofilariae) and temporarily impedes the release of such progeny by female macrofilariae, but a macrofilaricidal effect has been deemed minimal. Multiple doses of ivermectin may cumulatively and permanently reduce the fertility and shorten the lifespan of adult females. However, rigorous quantification of these effects necessitates interrogating longitudinal data on macrofilariae with suitably powerful analytical techniques.

**Methods:**

Using a novel mathematical modeling approach, we analyzed, at an individual participant level, longitudinal data on viability and fertility of female worms from the single most comprehensive multiple-dose clinical trial of ivermectin, comparing 3-monthly with annual treatments administered for 3 years in Cameroon.

**Results:**

Multiple doses of ivermectin have a partial macrofilaricidal and a modest permanent sterilizing effect after 4 or more consecutive treatments, even at routine MDA doses (150 µg/kg) and frequencies (annual). The life expectancy of adult *O. volvulus* is reduced by approximately 50% and 70% after 3 years of annual or 3-monthly (quarterly) exposures to ivermectin.

**Conclusions:**

Our quantification of macrofilaricidal and sterilizing effects of ivermectin should be incorporated into transmission models to inform onchocerciasis elimination efforts in Africa and residual foci in Latin America. It also provides a framework to assess macrofilaricidal candidate drugs currently under development.

## Teaser

We use a mathematical model fit to clinical trial data to estimate the efficacy of multiple-dose ivermectin regimens against onchocerciasis. We found marked macrofilaricidal activity of regimens used in routine mass drug administration, calling for revised projections on elimination timeframes.

In 1987 Merck & Co. Inc. committed to donate ivermectin for as long as needed to control human onchocerciasis. Subsequently, more than 2 billion treatments have been distributed in 29 countries throughout sub-Saharan Africa and in 6 countries in Latin America (http://www.merck.com/about/featured-stories/mectizan.html) by mass drug administration (MDA) programs. The Onchocerciasis Elimination Program for the Americas (OEPA) was launched in 1992 and the African Programme for Onchocerciasis Control (APOC) was launched in 1995 to coordinate these efforts, maintain high MDA coverage, and control onchocerciasis as a public health problem. Since release of the World Health Organization’s Roadmap on Neglected Tropical Diseases (NTDs) [1], the objective (in Africa) has been refocused toward elimination of the infection (OEPA aimed at elimination from the outset).

Barring the Amazonian onchocerciasis focus [2], elimination in Latin America is broadly on course [3]. In sub-Saharan Africa, where 99% of the infection cases occur, and notwithstanding some notable elimination successes [4–6], transmission is ongoing in many communities despite more than 15–20 rounds of annual (or 6-monthly) ivermectin treatments [7–10]. Addressing the question of whether elimination in Africa is feasible using current intervention strategies is a key issue. Before its closure in December 2015, APOC released guidance on alternative treatment strategies to accelerate progress toward elimination [11].

Mathematical modeling has demonstrated that the effectiveness and cost effectiveness of ivermectin MDA and the timeframes for elimination crucially depend on the long-term antifilarial action of ivermectin [12]. Ivermectin rapidly clears the skin-dwelling microfilarial progeny of *Onchocerca volvulus*, which is the filarial nematode that causes onchocerciasis, and temporarily prevents the release of new microfilariae by female worms. The dynamics of these so-called microfilaricidal and temporary sterilizing effects are well understood following a single standard ivermectin dose (150 μg/kg) [13]. However, because adult worms are long lived, with a life span of approximately 10 years [14], they are exposed to multiple ivermectin doses during an MDA program. Therefore, quantification of cumulative antifilarial effects on repeatedly exposed worms is essential for understanding the long-term epidemiological impact of MDA.

To date, cumulative antifilarial effects have been documented in an indirect manner (estimating their magnitude by fitting models to microfilarial trends following several treatment rounds), yielding conflicting results [15, 16]. However, direct observations on adult worm viability and reproductive status have not been linked to models for the underlying population biology of *O. volvulus*, a powerful approach that can translate clinical trial data into measures of drug efficacy [17]. Macrofilaricidal activity has been inferred from the smaller proportion of live worms extracted from participants given high-intensity regimens (frequency of more than twice per year; dose >150 μg/kg) compared to those given standard regimens [18] or not treated [19]. Similarly, fertility indicators from female *O. volvulus* exposed to high-intensity regimens are lower than those from females exposed to standard regimens, yet such indicators have not been translated into cumulative and potentially permanent effects of incremental exposures to ivermectin.

Here, we use our recently developed mathematical framework [17] to analyze longitudinal data on the viability and fertility of female adult *O. volvulus* collected from participants of the single largest and most recent randomized control trial, which was conducted in an endemic area of Cameroon [18] (a summary of other smaller and older trials is provided in the Supplementary Materials, Supplementary Figures 1–4 and Supplementary Table 1). We estimate the macrofilaricidal and antifertility activity of multiple ivermectin doses given at standard (annual) and high (3-monthly) frequencies and relate our findings to observations from previous studies on the antifilarial activity of multiple ivermectin doses and other macrofilaricidal therapies. We consider how these effects should be incorporated into transmission models to support onchocerciasis control and elimination and discuss how our findings can be used to assess the performance of candidate macrofilaricidal drugs.

## METHODS

### Trial Design and Data Collection

**Figure 1.**
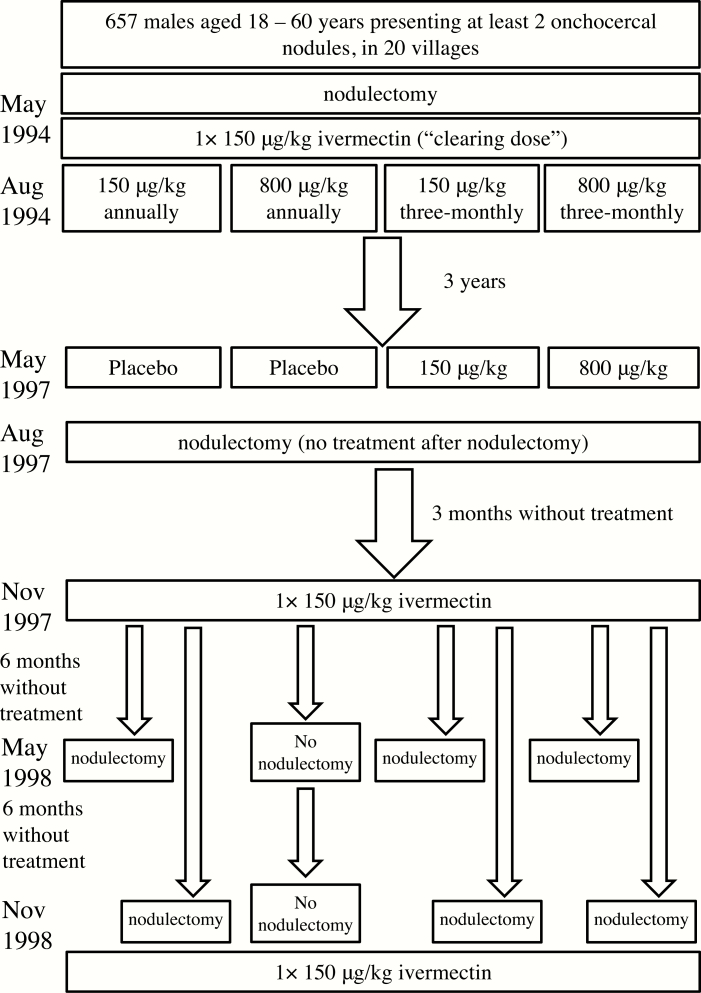
Schematic representation of the study design.

The data were collected between 1994 and 1998 during a clinical trial undertaken in a hyperendemic onchocerciasis focus with no history of ivermectin distribution or vector control and located in the Mbam River Valley, Central Region, Cameroon. The trial design is represented schematically in Figure 1; details are given in Gardon et al [18]. Briefly, 657 consenting males (to avoid contraindications associated with ivermectin in pregnancy), aged 18 to 60 years and with at least 2 palpable onchocercomas (subcutaneous nodules), were randomly assigned to 1 of 4 ivermectin treatment groups (Table 1). A single, randomly selected nodule was surgically excised from each participant on up to 3 occasions: prior to ivermectin treatment in May 1994; 3 years after the first trial dose in August 1997; and in either June 1998 or November 1998, 6 or 12 months after the last ivermectin treatment (Figure 1, Table 1). Following guidelines provided by Gardon et al [18], female worms within excised nodules were histologically classified (see Supplementary Materials) as nonfertile, *N*: live, nonfertile worms including potentially fertile females not currently inseminated, producing oocytes which transform into unfertilized ova and then degenerate as well as empty, senescent worms no longer producing oocytes; fertile, *F*: inseminated or re-inseminated and producing embryos of any stage up to live microfilariae; and moribund or dead, *D*: with few or no internal organs, collapsed body wall, and partial or total calcification. Data were analyzed per protocol rather than intent-to-treat; individuals who missed a treatment were systematically excluded from the cohort. We did not analyze data on male worms because their smaller size hinders their detection in histological sections and because very few were identified as dead or moribund [18]. This is common [20], probably because males tend to leave nodules to seek females [21] and may die outside of nodules.

**Table 1.** Summary of Trialed Ivermectin Treatment Regimens

Group	Dose	Frequency	Description^a^
1, Standard	150 µg/kg	Annual	3 × 150 µg/kg annually for 3 years
2, High dose	400–800 µg/kg	Annual	1 × 400 µg/kg and then 2 × 800 µg/kg for 2 years
3, High frequency	150 µg/kg	3-monthly	12 × 150 µg/kg 3-monthly for 3 years
4, High dose, high frequency	400–800 µg/kg	3-monthly	2 × 400 µg/kg and then 10 × 800 µg/kg for 2.5 years

^a^All individuals were additionally treated with a clearing dose of 150 µg/kg 3 months prior to the start of the study (immediately after the baseline nodulectomy) and with a final dose of 150 µg/kg 6 months after the final treatment.

### Modeling Approach and Statistical Inference

We used the treatment submodel embedded within our transmission model EPIONCHO [22, 23], originally developed to describe the population dynamics of adult female *O. volvulus* exposed to a single standard dose (150 μg/kg) of ivermectin [13]. We extended this model to track mean numbers of live and dead worms per host, in addition to the fertility (fertile or nonfertile) status of live worms, through different multiple-dose ivermectin treatment regimens. It was assumed that the (prior) life expectancy of (untreated) adult female *O. volvulus* was 10 years, with 95% of worms dead by age 14 years [14]. Mathematical details are given in the Supplementary Materials, a schematic in Supplementary Figure 5 and a list of parameter definitions in Supplementary Table 2.

The framework captures macrofilaricidal and sterilizing (temporary and/or permanent) effects, referred to collectively as antifilarial effects, on worms differentially exposed to ivermectin via parameters that increase the relevant population dynamics rates above that of unexposed worms (eg, an excess mortality rate; Supplementary Figure 6). These effects can vary with dose and frequency and can change dynamically such that the cumulative effects on viability or fertility can accrue in a constant decreasing or increasing manner with incremental exposures. Because of ongoing transmission, previously unexposed worms could have been acquired at any time during the trial. Therefore, we modeled explicitly the antifilarial effects on differentially exposed worms (Supplementary Figure 6) [13].

We modeled the temporally dynamic proportion of live female worms and the proportion of fertile (live) worms within hosts given each treatment regimen. By introducing host-specific random effects, we reconciled the modeled group-based dynamics with participant-specific longitudinal data, producing a statistical model to account for correlation among worm counts made repeatedly on the same individual, permitting (random) nonspecific variation among participants. We modeled state probabilities (proportions of live and fertile worms) to nullify the effects of variation in the incidence of new infections among participants (see Supplementary Materials for details). We did not use data on skin microfilarial densities [18] to avoid invoking additional assumptions on the production of microfilariae by female worms and on the competing dynamics of ivermectin’s microfilaricidal and sterilizing effects [13]. Hence, because the focus here is on antimacrofilarial activity, we used data on macrofilariae only.

We considered 4 models, model 0 to model 3, covering all combinations of antifilarial effects, with model 0 being the null model of no macrofilaricidal or permanent sterilizing activity of ivermectin but including a temporary sterilizing effect [13] (Table 2). We assumed that the first exposure to ivermectin does not exert any macrofilaricidal or permanent sterilizing effect [13], and we did not consider postulated prophylactic effects (Supplementary, Prophylactic effects of ivermectin). We fitted these models to the data in a Bayesian framework using Markov chain Monte Carlo techniques (see Supplementary Materials for details). We reflected a priori uncertainty in parameter values using prior distributions (priors). Parameters with information available in the literature were assigned informative priors, reflecting published uncertainties. These include the parameters that define the temporary sterilizing effect of a single standard dose of ivermectin [13], permitting permanent sterilizing effects to be resolved. Parameters without published information were assigned uninformative priors and include all parameters that govern the macrofilaricidal and permanent sterilizing activity (Supplementary Table 2).

### Transmission Intensity

Without data on transmission intensity during the trial (eg, numbers of infective larvae per blackfly) [18], we tested the robustness of the models to different assumptions on short-term (within year) and long-term (between year) changes in transmission. We fitted 4 variants (A to D) of models 0 to 3 (Table 2) covering combinations of short-term seasonal variation in transmission (mediated by fluctuations in the abundance of blackflies) and a long-term decline in transmission, perhaps induced by the trial (although this was unlikely; see Supplementary Materials for discussion). To capture seasonal variation, we fitted a sinusoidal function to data on blackfly numbers collected in the vicinity just before the trial (see Supplementary Materials for details). To capture long-term reductions in transmission, we modeled an exponential decline such that during the last year of the trial, the intensity of transmission was approximately half of its initial value (see Supplementary Materials and Supplementary Figure 7).

## RESULTS

### Antifilarial Indications and Population Dynamics

**Figure 2.**
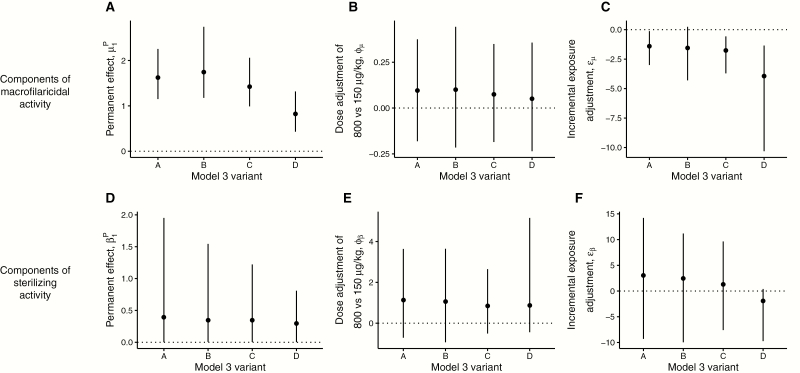
Summary of estimated parameters that define the antifilarial activity of multiple doses of ivermectin under different assumptions on the transmission dynamics during the trial. In each panel, the data points and vertical lines indicate the means and 95% Bayesian credible intervals (BCIs) of the posterior distributions estimated from variants A (constant short-term and long-term transmission), B (seasonal short-term transmission; constant long-term transmission), C (constant short-term transmission; declining long-term transmission), and D (seasonal short-term transmission; declining long-term transmission) of the best fitting model 3 (see Supplementary Table 3 for deviance information criteria). The horizontal dotted lines in each panel indicate the null (zero) effect of each parameter. Hence, parameters with BCIs that cross the dotted line can be interpreted as not statistically significantly different from zero. For example, in panels (A) and (D) none of the BCIs include 0, indicating statistically significant (permanent) macrofilaricidal and sterilizing activity of multiple-dose ivermectin regimens. By contrast, in panels (B) and (E) all BCIs cross 0, indicating no statistically significant effect of dose on either macrofilaricidal or permanent sterilizing activity. The parameter posteriors estimated from the model variants are generally similar and therefore robust to different assumptions on the transmission dynamics during the trial.

The best fitting models under all assumptions of seasonal variation and long-term declines in transmission include both macrofilaricidal activity and permanent sterilization (Supplementary Table 3), indicating that both processes operate. Model 3A had the lowest deviance information criterion (DIC) [24], indicating adequacy of a constant transmission assumption (Table 2). Parameter estimates are only modestly affected by different assumptions on seasonal variation and long-term declines in transmission (Figure 2; Supplementary Table 4).

**Table 2.** Models and Variants Incorporating Different Antifilarial Effects and Different Assumptions on the Transmission Dynamics During the Trial

Model	Permanent Antifilarial Activity	Variant	Assumptions on Short-term and Long-term Changes in Transmission
0	• No macrofilaricidal activity • No sterilizing activity	A	• Constant short-term transmission • Constant long-term transmission
1	• No macrofilaricidal activity • Sterilizing activity	B	• Seasonal short-term transmission • Constant long-term transmission
2	• Macrofilaricidal activity • No sterilizing activity	C	• Constant short-term transmission • Declining long-term transmission
3	• Macrofilaricidal activity • Sterilizing activity	D	• Seasonal short-term transmission • Declining long-term transmission

Nota bene: All variants A to D of models 0 to 3 were fitted to the data separately (ie, 16 separate fits in total).

**Figure 3.**
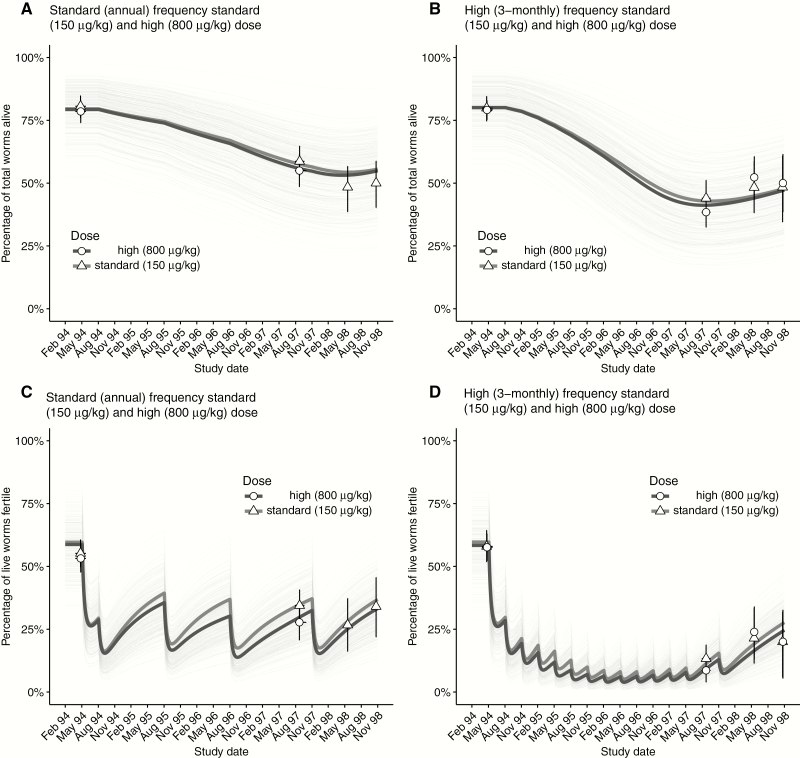
Dynamics of *Onchocerca volvulus* viability and fertility through multiple rounds of ivermectin treatment. Each panel depicts either the percentage of live female *O. volvulus* (panels A and B) or the percentage of fertile (live) female *O. volvulus* (panels C and D) exposed to ivermectin treatments given annually (panels A and C) or 3-monthly (panels B and D) at standard (150 µg/kg) and high doses (800 µg/kg). The thick gray and black solid lines indicate, respectively, the marginal mean (averaged across all hosts) dynamics for standard or high-dose regimens. The thin gray lines indicate the individual host dynamics mediated by the estimated random effects terms within the statistical model (see Supplementary Materials for statistical details). For clarity, no graphical distinction is given among individual hosts given either standard or high-dose regimens. In each panel, the triangular and circular markers denote, respectively, the observed percentages of live or fertile (live) females extracted from participants given either standard or high-dose regimens. Vertical error bars denote 95% confidence intervals that were calculated using a nonparametric weighted bootstrapping procedure to account for the variable number of female *O. volvulus* extracted from each host. Horizontal error bars (often narrower than the data point) indicate the range of nodulectomy dates at each sampling round.

Figure 3 shows the dynamics, estimated from model 3A, in the proportions of live and fertile female *O. volvulus* through the 4 multiple-dose treatment regimens (Table 1). Model 3A includes macrofilaricidal activity, permanent sterilization, and constant (seasonal and long-term) transmission (parameter estimates are depicted in Supplementary Figure 8). Depicted are both the mean dynamics among all trial participants given a particular regimen and the individual (host) dynamics. The observed (grouped) data are shown for visual appraisal of model fit.

### Macrofilaricidal Activity

The decline in the proportion of live worms (Figure 3A and 3B) is protracted and driven by the permanent excess (additional) mortality of live worms exposed to ivermectin compared to unexposed worms (Figure 2A). Macrofilaricidal activity is not significantly associated with dose (150 μg/kg vs 800 μg/kg; Figure 2B). The pace at which the macrofilaricidal effect accumulates declines with incremental exposures (Figure 2C negative incremental exposure adjustment for all model variants).

**Figure 4.**
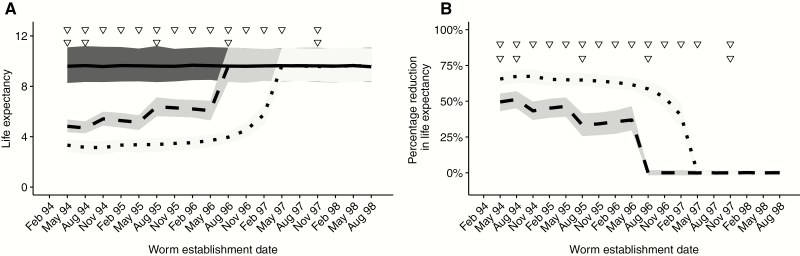
The life expectancy of adult *Onchocerca volvulus* multiply exposed to ivermectin. The dashed and dotted lines in panel A show the estimated life expectancy (in years) of adult female *O. volvulus* exposed to annual and 3-monthly treatments with ivermectin at a standard dose (150 µg/kg) (there is no substantive difference in the corresponding estimates for a high dose of 800 µg/kg dose, Figures 2 and 3). The solid line in this panel (*A*) depicts the estimated life expectancy of hypothetically unexposed worms. In panel (*B*), the dashed and dotted lines express the estimated life expectancies as percentage reductions compared to hypothetically unexposed worms. Estimates are plotted against the date of infection, that is, the time of establishment of female worms. Treatment dates with ivermectin are represented by the triangles: 3-monthly treatments on the top row and annual treatments below. Hence, worms that establish at an earlier date are exposed to more treatments and incur greater reductions in life expectancy. In each panel, gray shaded areas represent 95% Bayesian credible intervals. Life expectancies were calculated from the estimated parameter posteriors of model 3A (the best fitting model, see Supplementary Table 3 for deviance information criteria). Details of these calculations are given in the Supplementary Materials.

Figure 4 shows how the estimated life expectancy of female *O. volvulus* is reduced with increasing exposure to ivermectin (see Supplementary Materials for details of this calculation). Worms that establish in participants before the first treatment with ivermectin (and therefore are maximally exposed) have a life expectancy of approximately 4.8 years for the standard-dose annual regimen and 3.3 years for the 3-monthly regimen. This corresponds to 50% and 67% reductions compared to hypothetically unexposed worms, which have an estimated life expectancy of 9.6 years based on the life expectancy of *O. volvulus* in West African savannah [14].

### Sterilizing Activity

The modeled transient effects of the temporary sterilizing effect (Supplementary Figure 6) drives the oscillating proportion of fertile (live) worms (Figure 3C and 3D). The more protracted systematic decline is caused predominantly by the permanent sterilization of worms that are exposed to ivermectin on multiple occassions. Although this effect is estimated with substantial uncertainty (Figure 2D, models that included a permanent sterilizing effect (Table 2) were superior (according to the DIC) to those that omitted it (Supplementary Table 3). There is no statistically significant effect of dose on permanent sterilization (Figure 2E), but there is a nonsignificant tendency for the higher dose regimens to elicit a stronger effect. The pace at which the permanent sterilizing effect accumulates remains constant with incremental exposures (Figure 2F).

## DISCUSSION

Multiple doses of ivermectin have a partial macrofilaricidal effect and a modest permanent and irreversible sterilizing effect on female *O. volvulus* after 4 or more consecutive treatments, even at the annual frequency and 150 μg/kg dose used for MDA. A high 800-μg/kg dose does not significantly modify either effect. We used the scaffold of our newly developed modeling framework [17], linking longitudinal data collected at different follow-up times to the effects of ivermectin on fundamental parasite population processes. This was done in order to estimate the pronounced macrofilaricidal effect of 3 years of annual or 3-monthly multiple-dose regimens as, respectively, a 50% and 67% reduction in the life expectancy of female *O. volvulus*.

In their original analysis of these data, Gardon et al [18] identified a statistically significant higher proportion of dead female worms in patients who received the 3-monthly treatment compared to those treated annually. Raised proportions of dead worms from treated compared to untreated controls were also reported from trials in Liberia, Guatemala, and Sierra Leone (see Supplementary Materials and Supplementary Table 1 for a summary of these trials). The data in these studies were analyzed using traditional statistical techniques, making it impossible to translate proportions of dead worms (observed at variable follow-up times) into estimates of worm life expectancy. Moreover, with the exception of 9 treated participants from a trial in Guatemala [25], (Supplementary Table 1), none of the previous trials followed participants longitudinally.

Similarly, the original conclusions on the sterilizing activity of the multiple-dose regimens were based on comparisons of proportions of fertile worms among differently treated participants [18]. A lower proportion among 3-monthly compared to annually treated participants was reported; however, it was not possible to determine whether this was consistent with the (since well-described [13]) temporary sterilizing effect of ivermectin or indicative of irreversible impairment (a conundrum discussed elsewhere [26]). Our modeling approach resolves this and suggests a modest, permanent sterilization effect, albeit bounded by high uncertainty because both the macrofilaricidal and sterilizing effects influence observed proportions of fertile worms. The macrofilaricidal activity of ivermectin kills an increasing proportion of nonfertile worms before they become fertile for the first time, altering the worm population structure to comprise relatively more juvenile worms.

We recently used our modeling framework to quantify the macrofilaricidal activity of doxycycline, a tetracycline antibiotic that depletes the endosymbiotic *Wolbachia* bacteria essential to *O. volvulus* viability and fertility [17]. We reported an 80% reduction in the life expectancy of *O. volvulus* treated with 4–6 weeks of 100 mg or 200 mg daily doxycycline, which is higher than the macrofilaricidal efficacy of multiple doses of ivermectin estimated here. Other macrofilaricidal therapies include intravenous suramin, which is poorly tolerated with significant side effects and is therefore seldom used, and flubendazole, which was originally associated with serious intramuscular injection-site reactions [27] and subsequent development of an oral reformulation has been recently discontinued over toxicology concerns [28]. Hence, doxycycline is the de facto gold standard macrofilaricidal treatment for onchocerciasis, despite possible challenges associated with adherence to a relatively long treatment course.

This paucity of macrofilaricides emphasizes the need for new drugs to improve treatment of onchocerciasis in clinical settings and to bolster the armory of treatment options to assist with global control and elimination efforts. There is substantial and ongoing research into new drugs and drug combinations [29]. The analysis presented here, like that used to evaluate the efficacy of doxycycline, shows that the interpretation of clinical trial data is facilitated by population dynamics modeling techniques. These approaches can be used in a retrospective, analytical manner (as presented here) and prospectively to inform clinical trial design and the formulation of so-called target product profiles by simulating (parasitological or other) outcomes using postulated antifilarial activities.

The specific structure of the model used here is based on the treatment submodel embedded within EPIONCHO [22, 23], a transmission model that is used to explore the effectiveness of current and alternative intervention strategies that target onchocerciasis elimination in Africa [23, 30, 31]. In the future, to give our efficacy estimates more explicit programmatic interpretation, we will explore how partial macrofilaricidal and sterilizing effects of repeated rounds of ivermectin given during MDA alter current projections on the feasibility of achieving elimination targets [1, 32]. This will include revising projections on annual and 6-monthly treatment [30] and modeling the potential benefit of 3-monthly MDA regimens; the latter showing a significantly higher macrofilaricidal efficacy than annual MDA. Irrespective of our new understanding of the partial macrofilaricidal activity of repeated doses of ivermectin, maintaining high coverage and adherence to treatment, particularly in highly endemic areas, will remain extremely important to the effectiveness of MDA [22, 30].

Some epidemiological projections that incorporate an instantaneous macrofilaricidal effect of ivermectin have been made using the ONCHOSIM transmission model [22, 33]. These analyses attribute long-term reductions in microfilarial densities to either macrofilaricidal or permanent sterilizing activity, but not to both mechanisms acting simultaneously. Furthermore, their estimates of macrofilaricidal efficacy, based on clinical trial data from Guatemala [34] of 3-monthly treatments (Supplementary Table 1), rested on an assumption of no ongoing transmission during the trial and no resorption of dead worms [17, 35], risking underestimation of the true macrofilaricidal effect. Our approach untangles the permanent sterilizing and macrofilaricidal effects for both annual and 3-monthly ivermectin regimens at different doses and adjusts for the diluting effects of ongoing transmission.

Notwithstanding the partial macrofilaricidal activity of ivermectin, the search for new drugs to assist with the control and elimination of onchocerciasis remains extremely important [29]. Ivermectin cannot be used safely in loiasis–onchocerciasis co-endemic areas of West and Central Africa because of the risk of severe adverse events associated with high-grade *Loa loa* microfilaremia [36, 37]. Many communities in these areas are currently not served with effective onchocerciasis control. In such areas, macrofilaricidal (and permanently sterilizing) anti-*Wolbachia* therapies that do not affect *L. loa* (devoid of *Wolbachia*) are particularly promising. Anti-*Wolbachia* therapies may also impede parasite development in the blackfly vector [38], exerting a potential transmission-blocking effect. Elsewhere in Africa, reports of suboptimal responses to ivermectin continue [39] and should be considered an impetus to seeking alternative drugs to mitigate potentially emerging ivermectin resistance. Moxidectin is a promising candidate for MDA in these circumstances [40].

## CONCLUSIONS

We modeled longitudinal data from the most comprehensive clinical trial of multiple doses of ivermectin treatment using a powerful and contemporary population dynamics and statistical framework. We show that 4 or more consecutive treatments with ivermectin are partially macrofilaricidal and exert a permanent sterilizing effect on female *O. volvulus*, even at the standard dose and frequency used in routine MDA in Africa. It is essential to incorporate these effects into mathematical transmission models to reevaluate existing projections on the feasibility of eliminating onchocerciasis.

## Supplementary Data

Supplementary materials are available at *The Journal of Infectious Diseases* online. Consisting of data provided by the authors to benefit the reader, the posted materials are not copyedited and are the sole responsibility of the authors, so questions or comments should be addressed to the corresponding author.

## Supplementary Material

Appendix_revised

## Notes

***Acknowledgments.*** We thank J.A.N. Filipe for a preliminary modeling analysis of the data.

***Disclaimer.*** The funders had no influence on study design, collection, analysis, and interpretation of the data or the decision to submit the paper for publication.

***Financial support.*** This work was supported by the Wellcome Trust (grant 092677/Z/10/Z), the Neglected Tropical Disease Modelling Consortium (supported by the Bill & Melinda Gates Foundation in partnership with the Task Force for Global Health), and the United Nations Children's Fund/United Nations Development Programme/World Bank World Health Organization Special Programme for Research and Training in Tropical Diseases. The data were originally collected using funds from the River Blindness Foundation.

***Potential conflicts of interest.*** All authors: No reported conflicts of interest. All authors have submitted the ICMJE Form for Disclosure of Potential Conflicts of Interest. Conflicts that the editors consider relevant to the content of the manuscript have been disclosed.
