# Supplementary material for: Macrofilaricidal Efficacy of Repeated Doses of Ivermectin for the Treatment of River Blindness
Source: Clin Infect Dis. 2017 Jul 19;65(12):2026–34. doi: 10.1093/cid/cix616 (PMC5850622; doi:10.1093/cid/cix616)
Supplement: Appendix_revised [file cix616_suppl_appendix_revised.docx]

# The macrofilaricidal efficacy of repeated doses of ivermectin for the treatment of river blindness

Martin Walker, Sébastien DS Pion, Hanwei Fang, Jacques Gardon, Joseph Kamgno, Maria-Gloria Basáñez, Michel Boussinesq

# Appendix

## Review of multiple-dose ivermectin trials

We conducted a literature search using PubMed and Web of Science to identify other studies on the efficacy of multiple-dose ivermectin treatment regimens for treating onchocerciasis. We searched the keywords, abstract and title of studies in any language using the following: "(ivermectin OR heartgard OR sklice OR stromectol OR ivomec OR mectizan OR ivexterm OR scabo OR MK-933) AND (multiple dos$ OR repeated dos$ OR several dos$) AND (Onchocerc$ OR river blindness)”. We also searched the reference lists of any identified studies before applying the following inclusion criteria: (a) participants treated with at least 2 doses of ivermectin; (b) sufficient treatment information (e.g. number, frequency, follow-up time and dose), and (c) outcomes based on adult worm data. We did not include studies based solely on microfilarial data (larval progeny of adult worms) as part of the primary evidence base because of the increased (modelling) assumptions that such studies required to draw conclusions. Applying these criteria, we identified 3 separate clinical trials (excluding the study from which the data analysed in this paper were derived [1]) conducted in Guatemala [2-4], Liberia [5], and Sierra Leone [6-10], trialling from 4 to 12 treatments, of standard dose, given at frequencies ranging from every 2 weeks to annually (see Table 1 and Figures 1 to 4 of this *Appendix*).

These previous studies have exclusively evaluated simple outcome measures within relatively small groups of trial participants, such as the proportion of dead or non-fertile female worms, after multiple standard-dose treatment regimens. Only a single previous trial [3] (Figure 2 of this *Appendix*) has included participants longitudinally followed up over multiple rounds of treatment. We take a more powerful analytical approach that can: use data from individual participants followed up on multiple occasions (after different numbers of treatments); identify both killing (macrofilaricidal) and sterilising activity of standard and high dose/high frequency regimens; explicitly link observed trial outcomes to dynamic models for the underlying parasite population biology, and derive ivermectin-induced reductions in the life expectancy of adult *O. volvulus*.

## Parasitological methods

Each nodule was fixed in 20 times its volume of a mixture of 70% ethyl alcohol, 10% glycerol and 20% distilled water [11]. After 24 hours the fixative was renewed and the nodules were stored in the same fluid prior to sectioning. Nodules were halved or quartered across the most appropriate diameter depending on their size [12]. The nodules were embedded in paraffin wax, cut at a thickness of 6 µm, stained with haematoxylin and eosin and studied independently by Dr Brian O. L. Duke and one of the authors (M.B.). Differences of opinion were resolved by further study and discussion. While studying the slides, the observers were aware of which nodulectomy round the slides came from but they had no knowledge of the ivermectin dosage taken by the patients concerned.

## Population dynamics model

We modelled the population dynamics of *Onchocerca volvulus* using a series of coupled ordinary differential equations (ODEs) which describe rates of change in the number of non-fertile (*N_a,h_*), fertile (*F_a,h_*) and dead (*D_h_*) adult female worms in a nominal age compartment *a* exposed to *h* doses of ivermectin. This model is motivated by that presented by Basáñez et al. [13], who modelled the effects of single-dose ivermectin treatment. Here, however, we consider the population dynamics of moribund or dead worms—crucial for evaluating macrofilaricidal activity—and non-exponential adult parasite survival times [14]. That is, adult worms *N_a,h_* and *F_a,h_* must pass through *m* nominal age compartments before they die and pass into the moribund/dead, *D_h_* compartment. A further important feature of the model is that fertile worms can become non-fertile thereby capturing the reproductive biology of *O. volvulus* females who are periodically re-inseminated and undergo three to four reproductive cycles per year [15,16].

The stratification of worm populations into sub-populations exposed to different numbers of doses of ivermectin [17] is essential in capturing the dynamics of worm infection and mortality throughout the study period and in robustly evaluating the cumulative antifilarial effects of multiple doses of ivermectin. For example, letting *n* denote the maximum number of exposures to ivermectin for a particular treatment regimen (*viz*. *n*= 5 or *n*= 14 for the standard and high frequency regimens respectively, see Table 1 of the main text) some worms, acquired after the final treatment, will be unexposed to ivermectin (*h* = 0), while others present from the outset, and surviving the duration of the trial, will be exposed to the full *n* doses (*h* = *n*). The ODEs describing the dynamics within each nominal worm age compartment *a* are written succinctly as,

Table 1 Summary of cohorts included in multiple-dose trials of ivermectin collecting data on adult worms

| Treatment start year | Country | # Recruited participants | # Followed up participants | Ivermectin regimen | | | Follow up months after last treatment | # Nodules excised | # Female worms extracted | Reference |
| --- | --- | --- | --- | --- | --- | --- | --- | --- | --- | --- |
|  |  |  |  | # Treatments | Frequency | Dose |  |  |  |  |
| 1987 | Sierra Leone | NA^a^ | 18 | 4 | Six-monthly | 150 μg/kg | 6 | 35 | 135 | [6-8] |
|  |  | NA^a^ | 18 | 0^b^ | NA^b^ | NA^b^ | NA^b^ | 43 | 147 | [6-8] |
|  |  | NA^a^ | 19 | 5 | Six-monthly | 150 μg/kg | 1 | 32 | 84 | [6-8] |
|  |  | NA^a^ | 17 | 1^c^ | NA^c^ | 150 μg/kg | 1 | 35 | 130 | [6-8] |
|  |  | NA^a^ | 19 | 5 | Six-monthly | 150 μg/kg | 6 | 29 | 68 | [6-8] |
|  |  | NA^a^ | 15 | 1^c^ | NA^c^ | 150 μg/kg | 6 | 20 | 58 | [6-8] |
|  |  | NA^a^ | 25 | 5 | Six-monthly | 150 μg/kg | 18 | 54 | 195 | [6,7,9] |
|  |  | NA^a^ | 24 | 1^c^ | NA^c^ | 150 μg/kg | 18 | 66 | 241 | [6,7,9] |
|  |  | NA^a^ | 13 | 0^b^ | NA^b^ | NA^b^ | NA^b^ | 31 | 109 | [6,7,9] |
|  |  | NA^a^ | 36 | 10 | Six-monthly | 150 μg/kg | 9 | 79 | 179 | [6,7,10] |
|  |  | NA^a^ | 41 | 4 | Annual | 150 μg/kg | 9 | 79 | 264 | [6,7,10] |
| 1988 | Guatemala | NA^a^ | 40, 9 ^d^ | 1, 4^d^ | Six-monthly | 150 μg/kg | 6, 6^d^ | 65, 16^d^ | 84, 22^d^ | [3] |
|  |  | NA^a^ | 22, 38^d^ | 0, 0^b^ | NA^b^ | NA^b^ | NA^b^ | 45, 50^d^ | 67, 59^d^ | [3] |
|  |  | 12 | 9 | 4 | Three-monthly | 150 μg/kg | 4 | 16 | 22 | [4] |
|  |  | 12 | 8 | 8 | Three-monthly | 150 μg/kg | 4 | 14 | 20 | [4] |
|  |  | 12 | 11 | 11 | Three-monthly | 150 μg/kg | 4 | 18 | 22 | [4] |
|  |  | 12 | 9, 9, 10^d^ | 0, 0, 0^b, d^ | NA^b^ | NA^b^ | NA^b^ | 10, 12, 10^d^ | 18, 21, 23^d^ | [4] |
|  |  | 12 | 12 | 4 | Monthly | 150 μg/kg | 4 | 19 | 26 | [2] |
|  |  | 12 | 10 | 8 | Monthly | 150 μg/kg | 4 | 31 | 50 | [2] |
|  |  | 12 | 10 | 12 | Monthly | 150 μg/kg | 4 | 24 | 45 | [2] |
|  |  | NA^a^ | 46 | 0^b^ | NA^b^ | NA^b^ | NA^b^ | 48 | 78 | [2] |
| 1988 | Liberia | 30 | 29 | 6 | Two-weekly | 100 μg/kg | 4 | 82 | 168 | [5] |
|  |  | 30 | 25 | 0^b^ | NA^b^ | NA^b^ | NA^b^ | 102 | 198 | [5] |
| 1994 | Cameroon | 166 | 142, 46, 40^d^ | 4, 5, 5^d^ | Annually | 150 μg/kg | 12, 6, 12^d^ | 161^e^, 135, 45, 39^d^ | 353^e^, 317, 104, 94^d^ | [1] |
|  |  | 172 | 138 | 4 | Annually | 800 μg/kg | 12 | 159^e^, 129^d^ | 339^e^, 282^d^ | [1] |
|  |  | 161 | 135, 30, 34^d^ | 13, 14, 14^d^ | Three-monthly | 150 μg/kg | 3, 6, 12^d^ | 147^e^, 130, 40, 32^d^ | 310^e^, 293, 74, 62^d^ | [1] |
|  |  | 158 | 126, 42, 39^d^ | 13, 14, 14^d^ | Three-monthly | 800 μg/kg | 3, 6, 12^d^ | 144^e^, 117, 37, 37^d^ | 318^e^, 242, 80, 70^d^ | [1] |

^a^ Participants recruited retrospectively; ^b^ untreated controls; ^c^ singularly-treated controls; ^d^ participants from the same treatment group followed up longitudinally; ^e^ nodulectomy conducted at baseline, before treatment.

**Figure 1** Schematic of multiple-dose ivermectin trial conducted in Sierra Leone with first treatments given in 1987. Open arrows and open dotted arrows correspond to ivermectin doses of 150 μg/kg and placebo doses restectively. Black and open circles correspond to nodulectomies performed on treated and untreated participants respectively. The small vertical lines are spaced at intervals of 3 months. The numbers in the boxes correspond to, respectively, the number of participants who underwent nodulectomy, the number of extracted nodules and the number of adult female *Onchocerca volvulus* identified.

**Figure 2** Schematic of multiple-dose ivermectin trial conducted in Guatemala with first treatments given in 1988. Open arrows correspond to ivermectin doses of 150 μg/kg. Black and open circles correspond to nodulectomies performed on treated and untreated participants respectively. The small vertical lines are spaced at intervals of 3 months. The numbers in the boxes correspond to, respectively, the number of participants who underwent nodulectomy, the number of extracted nodules and the number of adult female *Onchocerca volvulus* identified.

**Figure 3** Schematic of multiple-dose ivermectin trial conducted in Liberia with first treatments given in 1988. Open arrows correspond to ivermectin doses of 150 μg/kg. Black and open circles correspond to nodulectomies performed on treated and untreated participants respectively. The small vertical lines are spaced at intervals of 3 months; treatments are every 2 weeks. The numbers in the boxes correspond to, respectively, the number of participants who underwent nodulectomy, the number of extracted nodules and the number of adult female *Onchocerca volvulus* identified.

$$\frac{dN_{1,h}\left(t\right)}{dt}=\frac{1}{2}\Delta_{h}\left(t\right)+[\beta_{0} + \beta_{1h}\left(t\right)\left]F_{1,h}\left(t\right) - \right[\alpha_{0} + \mu_{0} +\mu_{1h}\left(t\right)]N_{1,h}\left(t\right)$$

$$\frac{dF_{1,h}\left(t\right) }{dt}= \alpha_{0}N_{1,h}\left(t\right) - [\beta_{0} + \beta_{1h}\left(t\right) + \mu_{0} + \mu_{1h}\left(t\right)]F_{1,h}\left(t\right),$$

$\frac{dD_{h}\left(t\right)}{dt}=\left[\mu_{0} + \mu_{1h}\left(t\right)\right]\left[N_{m,h}\left(t\right) + F_{m,h}\left(t\right)\right] - \eta R_{h}\left(t\right),$ (1)

where function Δ*_h_*(*t*) describes temporal variation in the incidence of new infections (rate of incoming L3 larvae from biting blackflies and L4 establishing, with half assumed to be female); all other parameters are as defined in Table 2, and a schematic of the model is given in Figure 5 of this *Appendix*. To track the number of exposures to ivermectin received by each incoming cohort of new *O. volvulus* larvae, the incidence function Δ*_h_*(*t*) is modulated by a step function such that,

 (2)

where the annual transmission (*ATP*) is given by integrating over the continuous (and potentially seasonally varying) transmission potential (see *Seasonal variation in transmission*),

$ATP=\text{ }\int_{0}^{1} \lambda\left(t\right) dt$, (3)

and *δ*(*ATP*) captures density dependence in the probability of establishment of (immature) adult *O. volvulus* (which is parameterized in terms of the *ATP* rather than the instantaneous transmission potential, see *Density-dependent establishment of adult worms*). Parameter *τ_n_*_–_*_h_* in (2) denotes the time of treatment number *n*– *h* (remembering that *h* denotes the number of *exposures* to ivermectin and is *not* an indicator of the treatment number). Note that *h* takes integer values from 0 to *n* (*h* = 0, 1, …, *n*) and that *τ*_0_ is considered a special case, indicating the start of the model simulation, viz. *τ*_0_ = 0. Hence *h =* *n* describes the population dynamics of worms acquired *before* the onset of treatment and exposed to all *n* treatments. By contrast, and noting that *τ_n_*_+ 1_ is assigned an arbitrarily large value beyond the horizon of the simulation, *h*= 0 describes the population dynamics of worms acquired *after the last treatment,* and therefore unexposed to ivermectin. The total number of non-fertile, fertile and moribund worms at time *t*, *N*(*t*), *F*(*t*) and *D*(*t*) respectively, is the sum of the respective populations within nominal age compartment *a* = 1, 2, …, *m* exposed to *h* = 0, 1, …, *n* doses of ivermectin,

$$N\left(t\right) = \sum_{a} \sum_{h} N_{a,h}\left(t\right),$$

$$F\left(t\right) =\sum_{a} \sum_{h} F_{a,h}\left(t\right),$$

$D\left(t\right) = \sum_{h} D_{h}\left(t\right).$ (4)

**Figure 4** Schematic of multiple-dose ivermectin trial conducted in Cameroon with first treatments given in 1994. Open and black filled arrows correspond to ivermectin doses of 150 μg/kg and 800 μg/kg respectively. Black filled circles correspond to nodulectomies performed on treated participants. The small vertical lines are spaced at intervals of 3 months. The numbers in the boxes correspond to, respectively, the number of participants who underwent nodulectomy, the number of extracted nodules and the number of adult female *Onchocerca volvulus* identified.

The basic functional form of *β*_1_*_h_*(*t*) and *μ*_1_*_h_*(*t*) in equation (1) that is used to model cumulative embryostatic and macrofilaricidal activity respectively, is motivated by that used by Basáñez et al. [11] to model the dynamic sterilising activity of a single dose of ivermectin. Like Basáñez et al., we model an initial temporary spike in activity, *β*_1_^T^, followed by an exponential decay, *γ_β_*, but extending the Basáñez et al. model, we also include a residual permanent component to the sterilising effect, *β*_1_^P^ to capture the potential irreversible cumulative effects on female worm fertility of multiple exposures to ivermectin. We omitted a temporary component to the macrofilaricidal effect because preliminary analyses indicated that this parameter was not identifiable from the data. We, therefore, assumed only a permanent macrofilaricidal effect of each dose of ivermectin, *μ*_1_^P^_._ We further assumed that the first dose of ivermectin neither exerted a permanent embryostatic effect nor a (permanent) macrofilaricidal effect; that is, any permanent effects of ivermectin on female worms only begin to accumulate after their second exposure. The net (temporary and permanent) antifilarial effect on female worms is given by the summed activity of each dose of ivermectin in worm sub-populations exposed to *h* doses in total. Hence, the functions *β*_1_*_h_*(*t*) and *μ*_1_*_h_*(*t*) are written,

 (5)

and

 (6)

Here *I*(*t* – *τ_g_*) is a step function such that *I*(*t*– *τ_g_*) = 1 for *t* > *τ_g_* and 0 otherwise, and *τ_g_* denotes the time of the exposure *g* (out of the *h exposures* in total). Variable *x* is an indicator variable of a standard (*x* = 0) or high (*x*= 1) dose with coefficients *φ_β_* and *φ_μ_* which, respectively, modify the permanent components of the embryostatic and macrofilaricidal effects. That is, a high dose elicits a greater permanent embryostatic or macrofilaricidal effect for, respectively, *φ_β_* > 0 and *φ_μ_* > 0 and *vice versa*. The inclusion of exposure *g* (out of *h*) as a covariate alongside the coefficients ε*_β_* and ε*_μ_* permits the permanent components of, respectively, the embryostatic and macrofilaricidal activity at each dose to either increase (ε*_β_* > 0, ε*_μ_* > 0), decrease (ε*_β_* < 0, ε*_μ_* < 0) or remain constant (ε*_β_* = 0, ε*_μ_* = 0) in multiply exposed worms. These dynamics are illustrated in Figure 6 of this *Appendix.*

**Figure 5** The population dynamics model describes rates of change in the mean number of non-fertile (*N_a,h_*), fertile (*F_a,h_*) and dead (*D_h_*) adult female *Onchocerca volvulus* in nominal ‘age’ category *a* exposed to *h* doses of ivermectin (time dependency of state variables is omitted for brevity). The function Δ*_h_*(*t*) describes seasonal changes in the transmission potential and long-term changes in the annual transmission potential (both acting on the rate of incoming L3/L4 larvae from biting blackflies) and is modulated by a step function to track worm populations exposed to *h* doses of ivermectin. The functions *μ*_1_*_h_*(*t*) and *β*_1_*_h_*(*t*) describe, respectively, the macrofilaricidal and sterilising (including both temporary and permanent) effects of *h* doses of ivermectin (see Figure 6 in this *Appendix*). Function *μ*_1_*_h_*(*t*) defines the *per capita* excess mortality rate (above the natural background mortality rate of adult worms). The function *β*_1_*_h_*(*t*) defines the *per capita* excess rate of loss of fertility (above that associated with the natural cycle of fertile and non-fertile females). Other *per capita* rate parameters are defined as follows: *α*_0_, the rate of progression from non-fertile to fertile females; *β*_0_, the background (in the absence of ivermectin) rate of progression from fertile to non-fertile females; *μ*_0_, the background mortality rate of female worms, and *η*, the rate of resorption of dead worms.

Table 2 Parameter definitions, prior distributions and initial values

| **Parameter** | **Description**^a^ | **Prior limits**^b^ | **Prior distribution**^c^ | **Initial values**^d^ | **Reference** |
| --- | --- | --- | --- | --- | --- |
| *$\alpha_{0}$* | Per capita rate of progression from non-fertile to fertile worms | 0·51, 0·68^†^ | Gam(184, 3·2×10^-3^) | 0·59 | [11] |
| *$\beta_{0}$* | Per capita rate of progression from fertile to non-fertile worms | 0·23, 0·36^†^ | Gam(306, 1·1×10^-3^) | 0·33 | [11] |
| *$\mu_{0}$* | Per capita mortality rate of worms | 0·083, 0·125^‡^ | Gam(98, 1·0×10^-3^) | 0·1 | [12] |
| *$\eta$* | Per capita rate of resorption of dead worms | 0·4, 0·71^†^ | Gam(48, 1·1×10^-2^) | 0·53 | [18] |
| *$\beta_{1}^{T}$* | Temporary per capita excess rate of loss of fertility (magnitude of embryostatic effect) | 26·5, 40·9^†^ | Gam(85, 0·38) | 32·4 | [11] |
| ** | Adjustment to magnitude of embryostatic effect for high dose regimen | NA | Norm(0, 1·0×10^3^) | 0 | This work |
| *$\gamma_{\beta}$* | Rate of decay in magnitude of embryostatic effect | 15·9, 25·6^†^ | Gam(75, 0·26) | 19·6 | [11] |
| *$\beta_{1}^{P}$* | Permanent per capita excess rate of loss of fertility (magnitude of permanent sterilising effect) | NA | Gam(1·0×10^-3^, 1·0×10^3^) | 1·0×10^-3^ | This work |
| *$\varepsilon_{\beta}$* | Change in magnitude of embryostatic effect with increasing treatments | NA^e^ | Norm(0, 1·0×10^3^) | 0 | This work |
| *$\mu_{1}^{P}$* | Permanent per capita excess mortality rate (magnitude of macrofilaricidal effect) | NA | Gam(1·0×10^-3^, 1·0×10^3^) | 1·0×10^-3^ | This work |
| ** | Adjustment to magnitude of macrofilaricidal effect for high dose regimen | NA | Norm(0, 1·0×10^3^) | 1 | This work |
| *$\varepsilon_{\mu}$* | Change in magnitude of macrofilaricidal effect with increasing treatments | NA | Norm(0, 1·0×10^3^) | 0 | This work |
| *$\delta_{0}$* | Fraction of incoming worms maturing to adults when the annual transmission potential, ATP → 0 | 0·038, 0·15^†^ | Beta(2·69, 35·2) | 0·071 | [17] |
| *$\delta_{\infty}$* | Fraction of incoming worms maturing to adults when the annual transmission potential, ATP → ∞ | 0·00085, 0.0050 | Beta(7·75, 2577) | 0·0030 | [17] |
| *$c$* | Severity of the constraint acting on parasite establishment | 0·0018, 0·017^†^ | Gam(3·62, 1·6×10^-3^) | 0·0059 | [17] |
| *$\sigma_{1}^{2}$* | Variance among hosts in the proportion of live female worms | NA | Gam(1·0E^-3^, 1·0E^3^) | 1 | This work |
| *$\sigma_{2}^{2}$* | Variance among hosts in the proportion of (live) fertile female worms | NA | Gam(1·0E^-3^, 1·0E^3^) | 1 | This work |

^a^ Per capita rates are per adult female *Onchocerca volvulus.*

^b^ Published limits represent either a confidence (or a Bayesian credible) interval ^†^ or a range. ^‡^

^c^ Abbreviations: Beta, beta distribution defined by shape parameter 1 and 2 respectively; Gam, gamma distribution, defined by shape and scale parameters, respectively; Norm, normal distribution, defined by mean and variance, respectively. Informative priors were constructed by assuming that published parameters ranges fall at the 2·5 and 97·5 percentiles of the chosen prior distribution.

^d^ Where a reference is given, initial value represents published point-estimates.

^e^ Not available; where no prior information was available, vague / uninformative priors were defined.

## Seasonal variation in transmission

By default, the transmission potential, *λ*(*t*), in equation (2) was set to a constant. To test the influence of this assumption on the final statistical inference, we modelled seasonal (and long-term, see *Long-term trends in transmission*) variation in *λ*(*t*) as,

$\lambda\left(t\right) = \beta L\left(t\right)\frac{V\left(t\right)}{H},$ (7)

where *V*(*t*) is the seasonally-dependent density of the blackfly vector population; *H* is the (constant) density of the human host population; *β* is the per blackfly biting rate on humans, and *L*(*t*) is the mean number of infective L3 larvae per fly at time *t* [18]. Henceforth, and reflecting the much faster dynamics of the within-blackfly component of the parasite lifecycle, we assume that *L*(*t*) equilibrates instantaneously and remains constant, *L*^*^ [19], impervious to fluctuations in *V*(*t*) which cause small (damped) oscillations in the within-host adult parasite population [20].

We estimated *λ*(*t*) using data collected in the study location on the annual transmission potential and numbers of blackflies caught every month between April 1993 and March 1994, just before the start of the multiple dose ivermectin trial [21]. First, we fitted to data on the numbers of blackflies caught per month a functional form that has been used to capture seasonal changes in the population density of anopheline mosquitoes in Africa [22], and more recently to model seasonality in blackfly biting rates [23],

$V\left(t\right) = V^{MAX}_{ }\left\{b + \left(1 - b\right)\left[½\text{ }+\text{ }½ \cos\left(2\pi\left(t- u\right)\right)\right]^{\kappa}\right\}$ (8)

where *V^MAX^* is the peak density of the blackfly population. Second, we used the relationship

$ATP = \int_{0}^{1} \lambda\left(t\right) dt = \frac{\beta L^{*}}{H}\int_{0}^{1} V\left(t\right) dt$ (9)

to estimate the constant *βL*^*^/*H* from the fitted function *V*(*t*) and data on *ATP*. Finally, we estimated *λ*(*t*) using (7) and substituting *βL*^*^/*H* for *βL*(*t*)/*H*. We applied the same procedure to convert individual data points on numbers of blackflies caught per month to point estimates of transmission potentials. The smooth function *λ*(*t*) and the associated point-estimates from the blackfly data are depicted in Figure 7a of this *Appendix*. We capture uncertainty in *λ*(*t*) (and in the point estimates) using the estimated covariance of *V^MAX^* , *b*, *u* and *κ* which was approximated by the inverse of the Hessian matrix (the matrix of second order derivatives) estimated with a Newtonian optimization algorithm as part of the maximum likelihood estimation procedure.

**Figure 6** Schematic representation of modelled antifilarial activity of multiple doses of ivermectin. In each panel, the antifilarial activity of ivermectin is represented on the vertical *z*-axis as a per capita *excess* rate, plotted against time since the start of the study and the number of exposures to ivermectin received by different cohorts of infecting worms (acquired at different times) within study participants given five doses of ivermectin (as per the standard and high-dose regimens). Panels (a), (b) and (c) depict the macrofilaricidal activity under assumptions of incremental adjustments to the permanent effect of each additional exposure to ivermectin that either: (a) remains constant; (b) decreases, or (c) increases. Panels (d), (e) and (f), depict the analogous incremental adjustments to the (permanent) excess loss of fertility rate of adult female worms of each additional exposure to ivermectin that either: (d) remains constant; (e) decreases, or (f) increases. The model also captures dose-dependent (standard dose, 150 µg/kg or high dose, 800 µg/kg) adjustments to the antifilarial effects but for visual clarity these are not illustrated. Ribbons are plotted from the point that worms begin infecting participants in different time (exposure) intervals. For example, worms that infect participants after the penultimate treatment can be exposed to 1 dose of ivermectin only.

## Figure 1 Prior and posterior distributions of estimated parameters

## Long-term trends in transmission

Transmission was not measured during the multi-dose ivermectin trial [1] and although non-participants living in the same villages as the participants were treated annually, the geographical and therapeutic coverage were low and, therefore, it is unlikely that the treatments distributed during the trial impacted substantially on transmission. Nevertheless, to test the robustness of our results to a potential systematic reduction in the transmission potential during the trial, we modified equation (9) to include a long-term temporal trend component,

$ATP_{yr} =\int_{yr-1}^{yr} \lambda\left(t\right)\exp\left(-\upsilon t\right)_{.}$ (10)

Here, subscript *yr* denotes the year of the trial, such that *yr* = 1, 2, 3, 4, for the 4 years between 1994 and 1998. Hence, *ATP_yr_* is a discretized average of the transmission potential over the year, which varies seasonally and is expressed in continuous times. We considered a possible 50% reduction in the ATP between the first and last year of the trial as depicted in Figure 7b of this *Appendix*.

##

## Figure 7 Seasonal dynamics of the transmission potential (TP) and postulated long-term decline in the annual transmission potential (ATP) during the trial. The data points in (a) are derived from monthly data on the abundance of blackfly vectors (*Simulium squamosum*) in two communities in the Sanaga river basin close to where the multiple-dose trial of ivermectin was undertaken. These data were used to estimate TPs and a functional form was fitted that has previously been used to capture seasonal changes in the population density of anopheline mosquitoes and of blackfly biting rates (details are given in *Seasonal variation in transmission* above). Panel (b) illustrates the assumptions on seasonal variation in the TP (the total number of infective larvae potentially received per person per year at any given time in the year) and long-term changes in ATP; the average TP over the year, *viz*. the total number of infective larvae potentially received per person per year) used in the four variants of the population dynamics model (see Table 2 in the main text, variants A to D). The black solid and black broken lines indicate, respectively, seasonal variation in the TP and a constant ATP over the study period (note the dates on the *x*-axis). The grey solid and grey broken lines indicate, respectively, seasonal variation in the TP and a declining ATP over the study period such that the ATP at the end of the study is 50% of its starting value.

## Density-dependent establishment of adult worms

We modelled density dependence in the fraction of incoming larval *O. volvulus* successfully establishing as (immature) adult worms as a function of the *ATP* [18,19],

$\delta\left(ATP\right) = \frac{ \delta_{0} + \delta_{\infty}cATP}{1 + cATP}$ (11)

where *δ*_0_ and *δ*_∞_ are, respectively, the fractions of parasites maturing when the *ATP* → 0 and *ATP* → ∞, and *c* is a measure of the severity of the constraint acting on parasite establishment. These parameters have been estimated elsewhere [19] and we use these estimates and their associated uncertainties within the model (Table 2 of this *Appendix*).

## Prophylactic effects of ivermectin

A prophylactic effect of ivermectin against within-host incoming larval stages has been suggested from studies in chimpanzees by Taylor et al. [24] Conflicting evidence has been presented by Chavasse et al. [8] and Kläger et al. [9] (from data gathered during a trial conducted in Sierra Leone). Analysis of additional patient data from a previous trial also did not find evidence for a prophylactic effect [25]. More conclusive evidence has been presented for *O. ochengi* in calves [26]. It was not possible to incorporate such an effect within this framework with identifiability because of the absence of data on pre-adult larval stages. However, were such an effect to exist, it is important to note that it would probably reduce the magnitude of the estimated macrofilaricidal effect in a manner similar to the estimate from the models with a declining *ATP* – this would be the case because prophylaxis would, at least in the model, have a similar net effect to a declining incidence of new adult infections, analogous to the sensitivity analysis performed using the *ATP*.

## Statistical model

We used the population dynamics model in equation system (1) to derive probabilities (denoted Pr) that worms in participants given treatment regimen *i* observed at time *t_k_* after the first dose of ivermectin were alive, denoted *p_i_*(*t*), and—conditional on being alive—were fertile, denoted *q_i_*(*t*),

$$\Pr\left(alive\right) = p_{i}\left(t_{k}\right) = \frac{N_{i}\left(t_{k}\right) + F_{i}\left(t_{k}\right)}{N_{i}\left(t_{k}\right) + F_{i}\left(t_{k}\right) +D_{i}\left(t_{k}\right)}$$

$\Pr\left(fertile|alive\right) = q_{i}\left(t_{k}\right) = \frac{F_{i}\left(t_{k}\right)}{N_{i}\left(t_{k}\right) + F_{i}\left(t_{k}\right)}$ (12)

Note that *N_i_*(*t_k_*), *F_i_*(*t_k_*) and *D_i_*(*t_k_*) represent the mean number of non-fertile, fertile and moribund or dead worms (in groups of participants given a specific treatment regimen) and incorporates the net antifilarial effects of the differentially exposed worm sub-populations (within different nominal age compartments and exposure groups, see *Population dynamics model*).

We incorporated two random effects terms, denoted *b*_1_*_j_* and *b*_2_*_j_*, to render the group-level probabilities *p_i_*(*t_k_*) and *q_i_*(*t_k_*) specific to individual *j*, defining a pair of generalized additive mixed models,

$$\Phi\left[p_{ij}\left(t_{k}\right)\right] = \Phi^{-1}\left[p_{i}\left(t_{k}\right)\right] + b_{1j},$$

$$\Phi\left[q_{ij}\left(t_{k}\right)\right] = \Phi^{-1}\left[q_{i}\left(t_{k}\right)\right] + b_{2j}$$

$b_{1j} \sim Norm\left(0,\sigma_{1}\right),b_{2j} \sim Norm\left(0,\sigma_{2}\right).$ (13)

Here, Φ is the cumulative distribution function (and Φ^–1^ the inverse, quantile function) of the standard normal distribution, also called the ‘probit’ link function. We assumed that the random effects *b*_1_*_j_* and *b*_2_*_j_* are independently normally distributed among individuals on the (probit) link scale with standard deviation *σ*_1_ and *σ*_2_ respectively. This construction accounts for correlation among the data by modelling the repeated measures (on the same individual at different points in time) as independent, *conditional on* the individual-specific random effects terms [10].

We derived a likelihood for the data by considering the joint probability (Pr) of observing *y_ij_*(*t_k_*) live female worms and *z_ij_*(*t_k_*) fertile female worms, out of *n_ij_*(*t_k_*) females in total. This is given by the product Pr[*Y_ij_*(*t_k_*)] × Pr[*Z_ij_*(*t_k_*)|*Y_ij_*(*t_k_*)], analogous to the individual-based variant (i.e. after incorporation of the random effects terms *b*_1_*_j_* and *b*_2_*_j_*) of Pr[alive] × Pr[fertile | alive] given in equation (11). The likelihood of observations *y_ij_*(*t_k_*) and *z_ij_*(*t_k_*) (i.e. from individual *j* given treatment regimen *i* at time *t_k_*) is, therefore, given by the product of two binomial probabilities; or the log-likelihood by the sum of the logged probabilities,

$l_{ij}\left[y_{ij}\left(t_{k}\right),z_{ij}\left(t_{k}\right)|\mathbf{\theta}\right] = \ln f\left[y_{ij}\left(t_{k}\right)|n_{ij}\left(t_{k}\right),p_{ij}\left(t_{k},\mathbf{\theta}\right)\right] + \ln f\left[z_{ij}\left(t_{k}\right)|y_{ij}\left(t_{k}\right),q_{ij}\left(t_{k},\mathbf{\theta}\right)\right],$ (14)

where *f* denotes the binomial probability mass function and **θ** represents, in a generic fashion, the collection of model parameters defined in Table 2 of this *Appendix*. The likelihood of the entire dataset (described by the vectors **y** and **z**) is given by the sum of these individual-, time- and regimen-specific likelihood contributions,

$l\left(\mathbf{y},\mathbf{z}|\mathbf{\theta}\right) = \sum_{i} \sum_{j} \sum_{k} l_{ij}\left[y_{ij}\left(t_{k}\right),z_{ij}\left(t_{k}\right)|\mathbf{\theta}\right]$ (15)

## Parameter inference

We fitted the population dynamics model of equation system (1) in a Bayesian framework using Markov chain Monte Carlo (MCMC) techniques [27]. We defined informative prior distributions for parameters with estimates in the published literature—parameterized to reflect existing degrees of uncertainty—and uninformative or vague priors otherwise (Table 2 of this *Appendix*). We sampled from the (marginal) parameter posterior distribution (posteriors) using Metropolis or Gibbs sampling techniques for, respectively, the rate parameters of the population dynamics model (1) and the random effects terms of the statistical model in equation (13).

Specifically, we updated the population dynamics rate parameters individually using a normal proposal distribution and accepting proposed moves using the Metropolis criterion (invoking the symmetry of the normal proposal distribution). We dynamically refined the proposal variance of all parameters during the burn-in phase using the empirical running estimate of the variance of the Markov chain [28,29]. The differential equations of the population dynamics model (1) were solved for each proposed move to permit evaluation of the log-likelihood in equation (15) using a variable step size numerical integration algorithm implemented by calling C code from R [30] using the deSolve package [31].

We sampled the posteriors of the random effects (*b*_1_*_j_* and *b*_2_*_j_*) and standard deviations (*σ*_1_ and *σ*_2_) in 4 blocks using hierarchical Gibbs sampling techniques, conditioning on the current values of other parameters [27]. We sampled *b*_1_*_j_* and *b*_2_*_j_* using the latent variables approach for longitudinal binary probit models by Albert, Chib and Carlin [32, 33]. This approach exploits the conjugacy between the normally distributed random effects terms (*b*_1_*_j_* and *b*_2_*_j_*) and normally distributed latent variables that are simulated conditional on the observed data at each iteration of the Gibbs sampling algorithm (see Algorithm 4 in Chib *et al.* [33]). We sampled the posterior distributions of the variance parameters (that describe variability in the normally distributed random effects terms) by specifying uninformative conjugate gamma prior distributions with shape and scale parameters equal to 0.001 and 1000 respectively [27].

We wrote the Gibbs and adaptive-Metropolis sampling routines in C++ interfaced with R using the Rcpp package [34]. We initialized three starting values for the Markov chains to assess convergence on the parameter posteriors and to check that our conclusions were not sensitive to the choice of starting values [27]. The first 1,000 iterations of each chain were discarded as burn-in and a further 5,000 samples were used to estimate the posterior distributions. An example set of prior and estimated posterior distributions is given in Figure 8 of this *Appendix*.

**Figure 8** Parameter prior and estimated posterior distributions from the best fitting Model 3A (see Table 3 in this *Appendix*) that includes both permanent macrofilaricidal and sterilising activity and assumes that the transmission potential within a year is constant (no seasonality) and there are no long-term changes in the annual transmission potential (see Table 2 in the main text).

## Model comparisons

We developed several model parameterizations, each incorporating different assumptions on the short-term (within-year) transmission potential and the long-term (between-year) annual transmission potential (see Table 2 in the main text), with variant A assuming no change in transmission potential. We compared the parameter posteriors estimated from these models and the overall fit to the data using the Deviance Information Criterion (DIC) [35] (see Table 3 in this *Appendix*). The DIC is a Bayesian information theoretic approach that quantifies the trade-off between a model’s complexity and its fit to the data. The DIC values reported in Table 3 indicate general superiority of models incorporating macrofilaricidal and permanent sterilising activity (DIC values are lowest in Model 3 variants). They also indicate that the best (most parsimonious yet adequately fitting) of all models was Model 3A. This model incorporated no seasonal variation in blackfly (vector) abundance and no long-term decline in transmission. The parameter posteriors estimated from Model 3 variants A–D are summarised in Table 4 of this *Appendix*.

Table 3 Deviance information criterion (DIC)^a^ values of model variants incorporating different antifilarial effects and different assumptions on the temporal dynamics of transmission intensity during the trial

|  | Variant |  |  |  |
| --- | --- | --- | --- | --- |
| Model | A | B | C | D |
| 0 | 4663 | 4664 | 4663 | 4664 |
| 1 | 4653 | 4650 | 4653 | 4649 |
| 2 | 4657 | 4654 | 4647 | 4652 |
| 3 | **4609** | 4618 | 4621 | 4640 |

^a^ The deviance information criterion [35] (DIC) is a Bayesian information theoretic indicator that quantifies the trade-off between a model’s complexity and its fit to the data. A lower DIC (in bold) indicates a more parsimonious yet adequate fit.

## Life expectancy of adult worms

The population dynamics modelling framework—via the inclusion of multiple adult worm ‘age’ compartments—implicitly assumes that adult worm lifespans in the absence of treatment are gamma distributed with mean *μ*_0_^-1^ and variance *m*^-1^*μ*_0_^-2^ where *m* is the total number of nominal age compartments. Hence, it is straightforward to calculate the life expectancy (at establishment) of hypothetically unexposed worms (see Figure 4 in the main text) directly from the posterior distribution of *μ*_0_. The accumulating (dynamical) macrofilaricidal effects of repeated exposure to ivermectin render the distribution of worm life spans intractable and there exists no analytical expression for the life expectancy of worms subject to different numbers of exposures to ivermectin. Therefore, we estimated this quantity by simulating the progression of newly established worms through the stochastic version of the multiple adult worm age compartment model defined by equation (1). We used this simulation approach to record the time taken for worms to reach the dead compartment, *D_h_*, given a time of establishment in the first nominal (non-fertile) age compartment *N_1_*_,_*_h_* and subsequent exposures to ivermectin. By repeating the process many times, we estimated the distribution of worm life spans and the mean life span—the life expectancy—incorporating both stochastic uncertainty in progression process (progression between contiguous compartments was simulated as a heterogeneous Poisson process) and parameter uncertainty in the values of *μ*_0_ and those defining *μ*_1_*_h_*(*t*) in equation (6). The algorithm used to implement this approach is as follows:

1. Choose an establishment date for a cohort of newly acquired adult female *O. volvulus*.
2. Randomly sample values of *μ*_0_, *ε_μ_*, *φ_μ_* and *$\mu_{1}^{P}$* from their joint posterior distribution.
3. Simulate the progression of the cohort through the *m* nominal ‘age’ compartments as a heterogeneous Poisson process between contiguous compartments with rates of progression dependent on *μ*_0_ and *μ*_1_*_h_*(*t*).
4. Record the time taken for each worm to reach compartment *D_h_* and calculate the mean; this is an estimate of the life expectancy.
5. Repeat steps 2-4 1000 times to obtain a distribution of estimated life expectancies and calculate the mean and 95% interquartile range of this distribution to yield a point life expectancy estimate and an associated 95% Bayesian credible interval.
6. Repeat steps 1-5 for different establishment dates to estimate life expectancies associated with different numbers of exposures to ivermectin.

Table 4 Parameter posteriors under varying assumptions of the short-term (within-year) transmission potential (TP) and the long-term (between-year) annual transmission potential (ATP)

| **Parameter** | **Posterior mean; median (95% credible interval)** | | | |
| --- | --- | --- | --- | --- |
| ***Model 3*^a^** | **A** | **B** | **C** | **D** |
| *$\alpha_{0}$* | 0·63; 0·63 (0·56, 0·70) | 0·63; 0·63 (0·56, 0·70) | 0·62; 0·62 (0·56, 0·69) | 0·58; 0·59 (0·51, 0·65) |
| *$\beta_{0}$* | 0·33; 0·32 (0·29, 0·36) | 0·33; 0·33 (0·29, 0·36) | 0·33; 0·33 (0·29, 0·36) | 0·33; 0·33 (0·30, 0·37) |
| *$\mu_{0}$* | 0·10; 0·10 (0·09, 0·12) | 0·10; 0·10 (0·09, 0·12) | 0·11; 0·11 (0·09, 0·13) | 0·10; 0·10 (0·08, 0·12) |
| *$\eta$* | 0·51; 0·51 (0·43, 0·60) | 0·51; 0·51 (0·43, 0·59) | 0·55; 0·55 (0·45, 0·66) | 0·53; 0·53 (0·42, 0·66) |
| *$\beta_{1}^{T}$* | 23·97; 23·89 (19·47, 28·89) | 24·03; 23·91 (19·25, 29·20) | 24·28; 24·24 (19·71, 29·36) | 24·53; 24·50 (19·69, 29·65) |
| *$\gamma_{\beta}$* | 21·17; 21·08 (16·93, 26·00) | 21·16; 21·14 (17·09, 26·15) | 21·29; 21·18 (16·88; 26·38) | 20·31; 20·15 (15·93, 25·56) |
| *$\beta_{1}^{P}$* | 0·39; 0·20 (0·00, 1·95) | 0·35; 0·20 (0·01, 1·54) | 0·35; 0·25 (0·00; 1·22) | 0·30; 0·26 (0·00, 0·81) |
| ** | 1·13; 1·01 (-0·71, 3·64) | 1·06; 0·95 (-0·94, 3·65) | 0·84; 0·79 (-0·51, 2·65) | 0·87; 0·54 (-0·44, 5·16) |
| *$\varepsilon_{\beta}$* | 3·04; 2·81 (-9·30, 14·22) | 2·44; 2·61 (-9·96, 11·19) | 1·28; 1·11 (-7·60, 9·64) | -1·93; -0·89 (-9·74, 0·41) |
| *$\mu_{1}^{P}$* | 1·62; 1·59 (1·15, 2·26) | 1·74; 1·69 (1·18, 2·74) | 1·42; 1·39 (0·99; 2·06) | 0·82; 0·81 (0·43, 1·32) |
| ** | 0·07; 0·07 (0·01, 0·18) | 0·10; 0·10 (-0·22, 0·44) | 0·07; 0·08 (-0·19, 0·35) | 0·05; 0·05 (-0·24, 0·36) |
| *$\varepsilon_{\mu}$* | -1·40; -1·34 (-3·00, -0·12) | -1·55; -1·38 (-4·30, 0·24) | -1·77; -1·62 (-3·71, -0·57) | -3·94; -3·32 (-10·31, -1·34) |
| *$\delta_{0}$* | 0·07; 0·06 (0·01, 0·18) | 0·07; 0·07 (0·01, 0·18) | 0·09; 0·08 (0·02, 0·19) | 0·07; 0·07 (0·02, 0·16) |
| *$\delta_{\infty}$* | 0·00; 0·00 (0·00, 0·01) | 0·00; 0·00 (0·00, 0·01) | 0·00; 0·00 (0·00, 0·01) | 0·00; 0·00 (0·00, 0·01) |
| $c$ | 0·01; 0·01 (0·00, 0·01) | 0·01; 0·01 (0·00, 0·01) | 0·01; 0·01 (0·00, 0·01) | 0·01; 0·01 (0·00, 0·01) |
| *$\sigma_{1}^{2}$* | 0·33; 0·33 (0·26, 0·42) | 0·33; 0·33 (0·26, 0·41) | 0·33; 0·33 (0·26, 0·41) | 0·33; 0·33 (0·26, 0·41) |
| *$\sigma_{2}^{2}$* | 0·33; 0·32 (0·25, 0·42) | 0·33; 0·32 (0·25, 0·43) | 0·33; 0·32 (0·25, 0·43) | 0·32; 0·32 (0·25, 0·41) |

^a^ Model 3 includes both permanent sterilising and macrofilaricidal activity of multiple-dose ivermectin regimens and its variants make the following assumptions: A, a constant TP and ATP; B, a seasonal TP and a constant ATP; C, a constant TP and a declining ATP; D, a seasonal TP and a declining ATP.

## References

1. Gardon J, Boussinesq M, Kamgno J, Gardon-Wendel N, Demanga-Ngangue, Duke BOL. Effects of standard and high doses of ivermectin on adult worms of *Onchocerca volvulus*: a randomised control trial. Lancet, **2002**; 360: 203-10.
2. Duke BOL, Zeaflores G, Castro J, Cupp E, Muñoz B. Effects of multiple monthly doses of ivermectin on adult *Onchocerca vovlulus*. Am J Trop Med Hyg, **1990**; 43: 657-64.
3. Duke BOL, Zea-Flores G, Castro J, Cupp EW, Muñoz B. Comparison of the effects of a single dose and of 4 6-monthly doses of ivermectin on adult *Onchocerca volvulus*. Am J Trop Med Hyg, **1991** 45: 132-137.
4. Duke BOL, Zea-Flores G, Castro J, Cupp EW, Muñoz B. Effects of 3-month doses of ivermectin on adult *Onchocerca volvulus*. Am J Trop Med Hyg, **1992**; 46: 189-94.
5. Duke BOL, Pacque MC, Muñoz B, Greene BM, Taylor HR. Viability of adult *Onchocerca volvulus* after 6 2-weekly doses of ivermectin. Bull World Health Organ, **1991**; 69: 163-68.
6. Whitworth JAG, Morgan D, Maude GH, Downham DM, Taylor SW. A community trial of ivermectin on onchocerciasis in Sierra Leone: clinical and parasitological responses to the initial dose. Trans R Soc Trop Med Hyg, **1991**; 85: 92-6.
7. Whitworth JAG. Treatment of onchocerciasis with ivermectin in Sierra Leone. Parasitol Today, **1992**; 8: 92-6
8. Chavasse DC, Post RJ, Lemoh PA, Whitworth JAG. The effect of repeated doses of ivermectin on adult female *Onchocerca volvulus* in Sierra Leone. Trop Med Parasitol, **1992**; 43: 256-62.
9. Kläger S, Whitworth JA, Post RJ, Chavasse DC, Downham MD. How long do the effects of ivermectin on adult *Onchocerca volvulus* persist? Trop Med Parasitol, **1993**; 44: 305-10.
10. Kläger S, Whitworth JA, Downham MD. Viability and fertility of adult *Onchocrca volvulus* after 6 years of treatment with ivermectin. Trop Med Int Health, **1996**; 1: 581-9.
11. Schulz-Key H, Karam M. Quantitative assessment of microfilariae and adults of *Onchocerca volvulus* in ethanol-fixed biopsies and nodules. Trans R Soc Trop Med Hyg, **1984**; 78: 157-59.
12. Awadzi K, Opoku NO, Attah SK, Addy ET, Duke BOL, Nyame PK, Kshirsagar NA. The safety and efficacy of amocarzine in African onchocerciasis and the influence of ivermectin on the clinical and parasitological response to treatment. Ann Trop Med Parasitol, **1997**; 91: 281-96.
13. Basáñez MG, Pion SDS, Boakes E, Filipe JAN, Churcher TS, Boussinesq M. Effect of single dose ivermectin on *Onchocerca volvulus*: a systematic review and meta-analysis. Lancet Infect Dis, **2008**; 8: 310-22.
14. Plaisier AP, van Oortmarssen GJ, Remme J, Habbema JDF. The reproductive lifespan of *Onchocerca volvulus* in West African savanna. Acta Trop, **1991**; 48: 271-84.
15. Schulz-Key H, Karam M. Periodic reproduction of *Onchocerca volvulus*. Parasitol Today, **1986**; 2: 284-86.
16. Schulz-Key H. Observations of the reproductive biology of *Onchocerca volvulus*. Acta Leiden, **1990**; 59: 27-44.
17. Turner HC, Churcher TS, Walker M, Osei-Atweneboana MY, Prichard RK, Basáñez MG. Uncertainty surrounding projections of the long-term impact of ivermectin treatment on human onchocerciasis. PLoS Negl Trop Dis, **2013**; 7: e2169.
18. Basáñez MG, Boussinesq M. Population biology of human onchocerciasis. Phil Trans R Soc Lond B, **1999**; 354: 809-26.
19. Basáñez MG, Collins RC, Porter CH, Little MP, Brandling-Bennett D. Transmission intensity and the patterns of *Onchocerca volvulus* infection in human communities. Am J Trop Med Hyg, **2002**; 67: 669-79.
20. Walker M, Specht S, Churcher TS, Hoerauf A, Taylor MJ, Basáñez MG. The therapeutic efficacy and macrofilaricidal activity of doxycycline for the treatment of river blindness. Clin Infect Dis, **2015**; 60: 1199-1207.
21. Barbazan P, Escaffre H, Mbentengam R, Boussinesq M. [Entomological study on the transmission of onchocerciasis in a forest-savanna transition area of Cameroon.] [Article in French] Bull Soc Pathol Exot, **1998**; 91: 178-82.
22. Griffin J, Hollingsworth TD, Okell LC, *et al.* Reducing *Plasmodium falciparum* transmission in Africa: a model-based evaluation of intervention strategies. *PLoS Med*, **2010**; 7: e1000324.
23. Turner HC, Walker M, Attah SK, *et al*. The potential impact of moxidectin on onchocerciasis elimination in Africa: an economic evaluation based on the Phase II clinical trial data. Parasit Vectors, **2015**; 8: 167.
24. Taylor HR, Trpis M, Cupp EW, *et al*. Ivermectin prophylaxis against experimental *Onchocerca volvulus* infection in chimpanzees. Am J Trop Med Hyg, **1988**; 39: 86-90.
25. Boussinesq M, Chippaux JP. A controlled prospective trial of the prophylactic effects of a single dose of ivermectin against *Onchocerca volvulus*. Parasite, **2001**; 8: 255-59.
26. Tchakouté VL, Bronsvoort M, Tanya V, Renz A, Trees AJ. Chemoprophylaxis of *Onchocerca* infections: in a controlled, prospective study ivermectin prevents calves becoming infected with *O. ochengi*. Parasitology, **1999**; 118: 195-99.
27. Gelman A, Carlin JB, Stern HS, Rubin DB. Bayesian Data Analysis. 2^nd^ ed. London: Chapman & Hall, **2004**.
28. Haario H, Saksman E, Tamminen J. An adaptive Metropolis algorithm. Bernoulli, **2001**; 7: 223-34.
29. Roberts GO, Rosenthal JS. Examples of adaptive MCMC. J Comput Graph Stat, **2009**; 18: 349-67.
30. R Development Core Team. R: A language and environment for statistical computing. Vienna: R Foundation for Statistical Computing, **2011**.
31. Soetaert K, Petzoldt T, Setzer RW. Solving differential equations in R: package deSolve. J Stat Soft, **2010**; 33: 1-25.
32. Albert J, Chib S. Bayesian modeling of binary repeated measures data with application to crossover trials. In: Berry DA, Stangel DK. Bayesian Biostatistics. New York: Marcel Dekker, **1996**: 577-99.
33. Chib S, Carlin BP. On MCMC sampling in hierarchical longitudinal models. Stat Comput, **1999**; 9: 17-26.
34. Eddelbuettel D. Seamless R and C++ Integration with Rcpp. New York: Springer, **2013**.
35. Spiegelhalter DJ, Best NG, Carlin BP, van der Linde A. Bayesian measures of complexity and fit. J R Statist Soc B, **2002**; 64: 583-639.
